# Adaptive Filtering Method for Dynamic BOTDA Sensing Based on a Closed-Circuit Configuration

**DOI:** 10.3390/s26030789

**Published:** 2026-01-24

**Authors:** Leonardo Rossi, Gabriele Bolognini

**Affiliations:** Consiglio Nazionale delle Ricerche, 40129 Bologna, Italy

**Keywords:** distributed optical fiber sensors, strain and temperature measurement, dynamic temperature measurement, Brillouin Scattering, Brillouin optical time domain analysis, optical fiber sensors

## Abstract

A dynamic filtering system that can choose in real time between two different noise filters depending on the dynamics of the measured environment is presented. Unlike other adaptive filters approaches, this system does not require prior knowledge of the environment beyond noise characteristics. We implemented this system into a Brillouin optical time-domain analysis (BOTDA) sensing scheme using a closed-circuit control system for dynamic tracking of the Brillouin Frequency Shift (BFS) along the sensing fiber using a Proportional-Integral-Derivative (PID) controller. Through experiments and numerical simulations, we compare this method to the filtering capabilities of P and PI controllers chosen as optimal in a previous work for closed-circuit BOTDA (CC-BOTDA). Results show that the adaptive noise filter provides a dynamic response comparable to the other controllers, while increasing noise suppression by a factor between 30% and beyond 100%, showing how an adaptive system can improve suppression with only knowledge of the measurement noise.

## 1. Introduction

Over the past three decades, Brillouin optical time-domain analysis has been under a great deal of study due to its capability of exploiting the process of Stimulated Brillouin Scattering (SBS) [1,2,3] to monitor temperature and strain distributions over several tens of kilometers of optical fibers. In standard BOTDA schemes, the pump-probe frequency shift is swept over a range in the order of several hundreds of MHz with a step in the order of 1 MHz to reconstruct the Brillouin Gain Spectrum (BGS) to extract the Brillouin Frequency Shift (BFS) distribution [4,5]. Due to this process, classical BOTDA sensing is usually time-consuming, requiring several minutes per measurement. As a result, BOTDA is restricted to static and quasi-static measurements.

In recent years, growing interest in structural health monitoring has led to an increase in research for the development of dynamic strain and temperature measurement techniques, motivating multiple attempts to develop BOTDA schemes where the measurement time is reduced to less than a second, such as fast BOTDA (F-BOTDA) systems [6], systems with reduced or no frequency sweeps [7,8,9], or the slope-assisted BOTDA (SA-BOTDA) technique, which particularly allowed for measurements with frequencies in the hundreds of Hz [10,11,12,13,14,15]. One of the main limitations of this technique is that the measurement range for temperature and strain is limited to the width of the BGS where the relation between frequency detuning and the Brillouin Gain is linear, which is roughly equal to a few tens of MHz of BGS shift, equivalent to a few tens of °C or a few hundreds of με.

Several variations in the original SA-BOTDA scheme have been proposed to improve the measurement range [16,17,18,19], and while these methods were shown to be effective, they still increase the measurement range by a limited amount. In addition, measurements were only showcased for sensing fiber lengths of at most 100 m.

In [20], a different approach based on a closed-circuit (CC-BOTDA) configuration was proposed. In this method, an arbitrary waveform generator (AWG) was used to actively track the BFS changes along the sensing fiber, effectively extending the measurement range indefinitely with no frequency scanning involved. The active tracking of the BFS evolution was enabled by a proportional-integral controller (PI controller), whose response in terms of frequency correction for the pump-probe frequency shift was also used as the output of the measurement. In addition to these functions, the PI controller also acted as a low-pass filter, providing the ability to suppress noise in exchange for a slower dynamic response. It should be noted that, while acting as a filter, the PI controller was not directly applied to the probe trace and thus has no effect on its quality. This approach was further developed in [21] to compensate probe power fluctuations due to different modulation frequencies.

The filtering of data and removal of noise is an important factor in dynamic sensing: when dealing with a continuous stream of time-domain measurements, additional aspects beyond noise suppression, such as response speed and the capability of functioning in real time, become relevant, and the choice of filter has the potential of significantly boosting sensor performance. Alongside simpler filtering approaches, such as standard and exponential moving average, other filters exist whose features can be optimized through a specific criterion (such as maximum a posteriori probability or mean square deviation), as is the case for Weiner or Kalman filters [22]. These filters either require spectral characteristics or autocorrelation functions of the signal and noise (in the case of the Wiener filter), or a model of the evolution of the measurand (in the case of Kalman filters), or knowledge of the variance of the signal being detected. These requirements are usually too strict for BOTDA sensors, where few assumptions can be generally made on the quantities being measured, and a model of their evolution cannot be assumed to be available, since the sensor might be employed in a great variety of different applications. In principle, in these conditions, simpler filters remain as the only viable option, which are bound to the fundamental tradeoff between noise suppression and dynamic response (which is important to preserve the evolution of a time-domain signal) [23]. It should be noted that, although low-pass filters are expected to introduce a trade-off between noise suppression and dynamic response, their response time will be much faster compared to other typical limiting factors in static BOTDA schemes (e.g., due to frequency sweep rate constraints during BFS acquisition).

In this work, we present an approach overcoming this tradeoff by categorizing the behavior of the measured quantity in specific user-defined phases and applying filters that are more preferable for these phases, creating a real-time output (i.e., based only on previous and present measurements) that can have either a fast dynamic response or high noise suppression depending on the state of the measurand. The system achieves this by using two types of filters, a faster one (less noise suppression, better dynamic response) and a slower one (higher noise suppression, worse dynamic response). To decide which filter to apply, the system only requires the recent measurement history and data gathered from acquisition where the measurand was considered stable (e.g., constant temperature or strain), without requiring information on the spectral characteristics of the signal, its variance, or a model to predict the evolution of the system. By linking its behavior to data gathered in a stable phase, the system thus ensures consistent behavior at different noise levels. This is the case, for instance, of measurements at longer fiber distances (i.e., lower signal-to-noise ratio), where the noise reduction factor of the adaptive filter will remain unchanged.

We implement this system in an experimental setup similar to [20,21] and compare the results to the systems that were found to be best performing in that work, showing that we can achieve the same response speed while having up to 100% improved noise suppression when the signal is stable.

This work is structured as follows. In the remaining parts of this section, we evaluate the closed-circuit system by noting that with a P controller we can simply follow and directly acquire the temperature evolution, which can then be digitally filtered by our approach. In Section 2 (“Materials and Methods”), we describe the design of our system and the experimental application, involving dynamic temperature monitoring over a fiber length of 1 km and a measurement time of 160 ms. In Section 3 (“Results”), we present the results: compared to the filtering methods shown in [20], the presented adaptive filter shows increases of up to 30% in noise suppression when the faster filter is applied (roughly corresponding to situations where the temperature around the sensing fiber is changing) and 100% higher noise suppression when the slower filter is used instead (corresponding to situation where the environment around the fiber is stationary), while keeping the same response speed. In Section 4 (“Discussion”), we use a numerical simulation to provide a clearer view on the effect of noise suppression at different phases of the measurement process.

### Closed-Circuit BOTDA Working Principle

**Figure 1:**
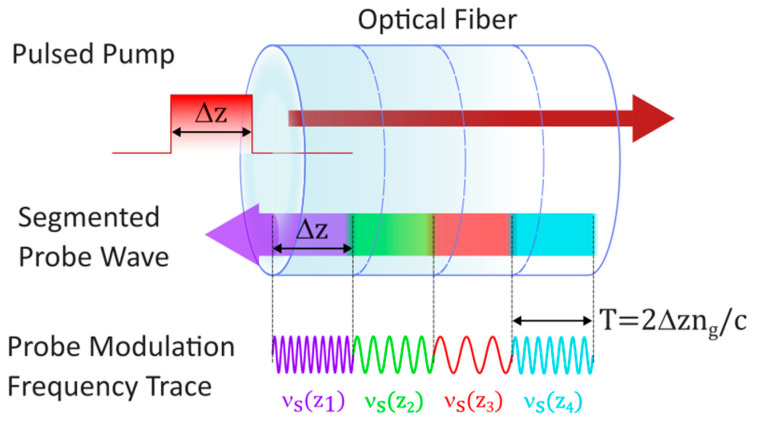
Tailoring of the probe frequency in CC-BOTDA. The probe modulation signal is divided into N segments corresponding to the length of the probe pulse, each one with its own modulation frequency.

The working principle of the CC-BOTDA consists of frequency modulation of the probe signal as shown in Figure 1, so that at every position z_i_ of the fiber the pump pulse overlaps with a counterpropagating probe wave at a different frequency ν_s_(z_i_). These variable probe frequencies ensure that SBS at every point of the fiber takes place with a different pump-probe frequency shift, which is constantly updated to make sure the Brillouin Gain is kept at set values of g_op_(z_i_), which depend on position but remain constant over time.

The pump pulse occupies at a given time a length of fiber equal to Δz, corresponding to a pulse duration of $T=2∆z\cdot n_{g}/c$, where $c/n_{g}$ is the group velocity. Every single segment of the probe modulation will have the same duration T and will thus correspond to a segment of fiber of length Δz.

**Figure 2:**
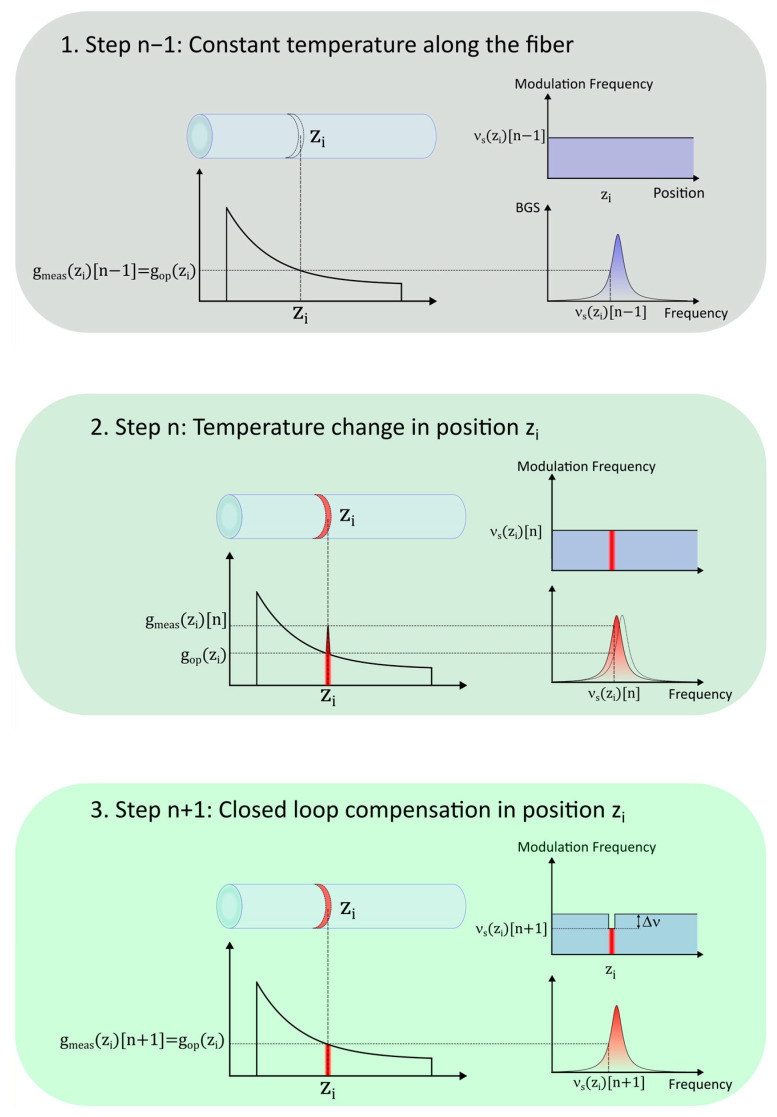
CC-BOTDA frequency tracking and measurement loop. At step n, there is a shift in the BFS, resulting in a change in gain from the setpoint g_op_(z_i_), which is then compensated by the closed-loop controller through a frequency shift Δν calculated from the change in gain.

The tracking method, illustrated in Figure 2, uses a PI controller to calculate the required change in frequency as a function of the measured gain. If at measurement step n and position z_i_ a shift in the BFS causes the gain to change from g_meas_(z_i_) [n−1] = g_op_(z_i_) to a new value g_meas_(z_i_) [n], the new frequency shift ν_s_(z_i_) [n] for the segment will be changed by an amount Δν defined by the PI controller and be set to the following:

(1) $$\nu_{s}\left(z_{i}\right)\left[n\right]=\nu_{s}\left(z_{i}\right)\left[n-1\right]+∆\nu =\nu_{s}\left(z_{i}\right)\left[n-1\right]-K_{P}\frac{g_{meas}\left(z_{i}\right)\left[n\right]-g_{op}\left(z_{i}\right)}{\eta }-K_{I}\sum_{k=1}^{n}\frac{g_{meas}\left(z_{i}\right)\left[k\right]-g_{op}\left(z_{i}\right)}{\eta },$$

where η is the slope of the linear region of the BGS, while K_I_ and K_P_ are the parameters of the PI controller.

As long as the set frequency shift stays within the linear region of the BGS, Equation (1) can be rewritten purely in terms of probe frequency correction, where ν_meas_(z_i_) is the new “correct” frequency that should be set given the gain measurement g_meas_(z_i_) [n]:

(2) $$\nu_{s}\left(z_{i}\right)\left[n\right]=\nu_{s}\left(z_{i}\right)\left[n-1\right]+K_{P}\left(\nu_{meas}\left(z_{i}\right)\left[n\right]-\nu_{s}\left(z_{i}\right)\left[n-1\right]\right)+K_{I}\sum_{k=1}^{n}\left(\nu_{meas}\left(z_{i}\right)\left[n\right]-\nu_{s}\left(z_{i}\right)\left[n-1\right]\right)$$

The ν_s_(z_i_) [n] sequence can then be taken as the measurement of the BFS for every position z_i_.

When, on the other hand, K_P_ = 1 and K_I_ = 0, ν_s_(z_i_) [n] is identical to ν_meas_(z_i_) [n], the control loop outputs the measured BFS without any kind of filtering, simply following its evolution. It is easy to see from this that the combined functions of tracking the evolution of the BFS and filtering the output sequence could also be achieved by employing a closed-circuit control with K_P_ = 1 and then applying a digital filter to its output. This way, it is possible to implement more complex filtering approaches while retaining tracking. It should be noted that the system’s tracking capabilities are affected by the choice of K_P_ and K_I_ parameters, especially for what concerns the circumstances that might cause the close-circuit system to fail to keep ν_s_(z_i_) inside the linear region. In particular, if K_P_ = 1, the tracking might fail if the measurement noise is high enough that random fluctuations will cause the frequency to be set outside the linear area of the BGS. If, on the other hand, K_P,_ K_I_ < 1, the set frequency will follow the measured frequency with a delay and will be less responsive to noise, but a sufficiently fast and sustained change in temperature or strain could cause the set frequency to drift outside of the BGS. Both of these circumstances can be considered extreme: for K_P_ = 1, the detection noise for the BFS has to be in the tens of MHz, equivalent to tens of °C or hundreds of με which would be an unusually high level of noise for most practical applications. For K_P_, K_I_ < 1, it would mean a shift of 10 s of MHz over few measurement cycles, which would imply an extreme change in temperature or strain over the course of a few seconds, which is uncommon in most BOTDA applications. As a consequence, outside of these specific circumstances, the choice of K_P_ = 1 can be assumed to not affect tracking.

As said above, using K_P_ = 1 for the closed-loop controller means that we can acquire the BFS evolution directly which can then be filtered by any type of digital filter, without being limited to the filtering capabilities of PI controllers for noise suppression. In the following sections, we will provide an example of an approach that, depending on the measurement history, will dynamically adapt in real time to adopt the preferred tradeoff between noise suppression and response time for time-domain signals.

## 2. Materials and Methods

### 2.1. Filter Design

In BOTDA applications, it is not always obvious to discern which feature is more important between noise suppression and dynamic response in the filtered measurement output, and it might actually be desirable to change the nature of the filter in real time as the situation being monitored changes. We present a filtering method for the following scenario: the measured system alternates in time between a “stationary” state, where the strain/temperature conditions remain mostly constant, followed by a “dynamic” state, where the strain/temperature can change significantly over a given timescale. We need to both maximize the measurement accuracy for the stationary states (employing a filter with a slower response) while also capturing dynamic states (employing a faster-response filter). To achieve this, the system must be capable of distinguishing actual changes in the environment from random noise and then employing the correct filter. In terms of real applications, such a situation could occur, for instance, while monitoring and characterizing cyclic thermal processes, which can take place in a variety of industrial environments.

### 2.2. Detection of Changes in Behavior

**Figure 3:**
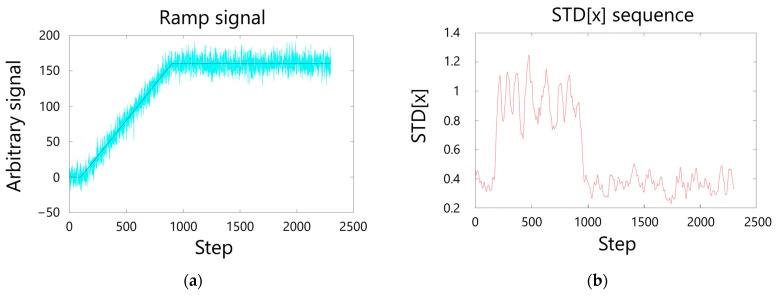
Example of arbitrary signal (blue line) with simulated noise (cyan line) (**a**) and corresponding STD[x] sequence, computed as the standard deviation of the last 50 points before point x (**b**).

To detect changes in signal behavior, we monitor it and then calculate the STD [n] sequence, defined as the standard deviation of the last X points, where the choice of X determines the overall sensitivity of the system. Figure 3 shows an example of a noisy signal (Figure 3a) and the corresponding STD [n] sequence with X = 50 (Figure 3b): STD [n] spikes in correspondence to the ramp, while the outside of the ramp [the STD sequence] settles on a baseline that depends on the level of noise.

To determine this baseline, a reference STD sequence can be acquired when the measurand is considered to have a constant value (i.e., constant temperature or strain in the case of BOTDA). From this sequence, we can extract the average $\bar{{STD}_{flat}}$ and the standard deviation $\sigma ({STD}_{flat})$ which together indicate how STD [n] changes when it is only affected by noise.

STD [n] can now be used to define transitions between stationary and dynamic states by observing the average of the last Y points of the STD [i] sequence. If it is greater than $\bar{{STD}_{flat}}+3\sigma ({STD}_{flat})$, then the changes in the sequence are caused by a source other than random noise and the state is considered to have shifted from static to dynamic.

If the state is dynamic and the average of the last Z points of the STD [i] sequence is lower than $\bar{{STD}_{flat}}$, the state is considered to have shifted from dynamic to static.

The choice of the X,Y,Z parameters is arbitrary and determines how easily the system will transition from one state to another and can change depending on noise and expected evolution. In this work, we have chosen X = 15, Y = 50, and Z = 60.

It is to note that while there is still a need for some a priori knowledge of the measurement environment, particularly in terms of the noise floor and in the choice of the parameters, these requirements only rely on general knowledge of the range of expected fluctuation of temperature and strain with respect to noise and are overall less strict than knowledge of the model of its evolution or of the variance of the signal.

It should also be emphasized that one of the strongest advantages of this method is that it can be applied independently to every single sensing point, meaning that each point can be sensed in a different state (stationary/dynamic) depending on its specific local environment.

In addition, this approach allows the thresholds for transitions between phases to automatically depend on the noise level, meaning that, for instance, sensing points at longer fiber distances will automatically result in higher thresholds due to increased noise levels, reflected in their respective $\bar{{STD}_{flat}}$ and $\sigma ({STD}_{flat})$ values, which will provide consistent behavior, e.g., along different distances along the fiber.

**Figure 4:**
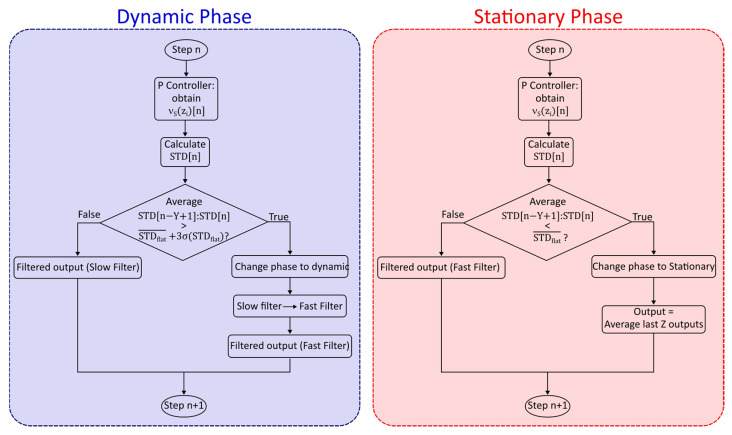
Flowchart representation of the adaptive filtering process at a given position z_i_ and measurement step n, showing the different behaviors when the system is in a dynamic or stationary phase.

A flowchart description of this process (also including elements descripted in Section 2.4) is shown in Figure 4.

### 2.3. Choice of Stationary and Dynamic Filters

As stated before, we want to use a filter with faster response speed when the system is in a dynamic phase and a filter with higher noise suppression in the stationary phase. For this work, we will take as templates the filters that were employed in [20], defined by Equation (2).

**Figure 5:**
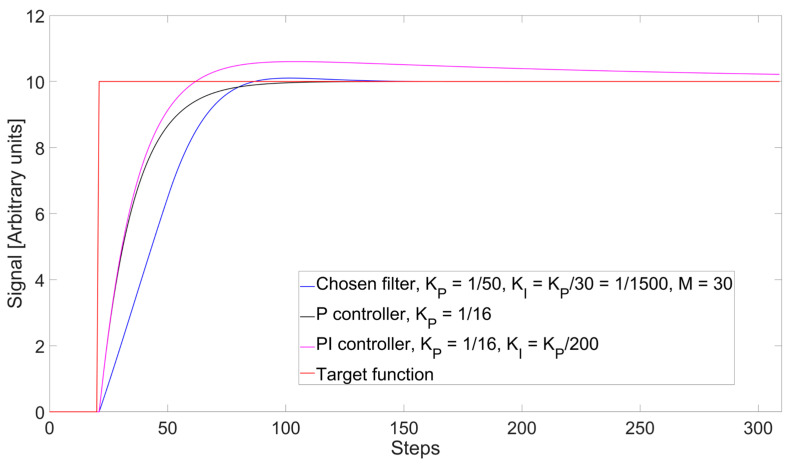
Comparison between the step responses of the chosen limited PI filter and the P and PI controllers employed in [20].

The dynamic filter we will employ for this work is a variant of the PI controller, where we apply a limit on the number of errors in the sum of the integral component in order to prevent an overshoot effect with large changes in setpoint that was noted in [20]. The formula to calculate each new element of the $\nu_{s}\left(z_{i}\right)\left[n\right]$ sequence is the same as Equation (2), but the sum is limited to the elements from *n* to *n*-*M*. By choosing the correct parameters, this version of the filter equivalent to a PI controller can achieve a significantly lower overshoot compared to the standard counterpart. For this work, we have chosen K_P_ = 1/50 and K_I_ = 1/1500 and M = 30. To show why we chose these parameters, in Figure 5 we present a comparison of the dynamic response to a step function for this filter and the ones that were used in [20], that is, a P controller with K_P_ = 1/16 and a PI controller with K_P_ = 1/16, K_I_ = K_P_/200. As can be seen, the filter we chose reaches the top of the step function slightly faster than the others, with only a minimal overshoot of 1% of the maximum value, while the PI controller has an overshoot of 5%. In addition, by adding Gaussian noise to the simulation and taking the standard deviation of 200 points after the step for the filtered signals, it is possible to estimate their noise reduction factor as 7.2, 5.4, and 5.2, respectively, for our filter, the P controller, and PI controller, showing how we can already obtain more than 30% improved noise suppression, without significant losses in dynamic response with the parameters we have chosen.

For the stationary filter, on the other hand, we opted to choose one equivalent to a P controller with a small P parameter to ensure a high degree of noise suppression and a slow dynamic response. For this work, we have chosen K_P_ = 1/80 and K_I_ = 0. It is important to note that these are not the only possible choices, and better results could be obtained with different filters.

### 2.4. Transition Between Filters as the Phase Changes

To ensure continuity of the system output, it is important to define what happens when the system transitions between different phases. For what concerns the transition from stationary to dynamic, it is simply possible to change the filter parameters. For a transition from dynamic to stationary, on the other hand, especially if filters analogous to P controllers are used, simply changing the parameters might introduce a mismatch between the measured and real value, which will introduce a delay as the filter settles into the new value, similar to the response time needed after a step-like change. To prevent this, upon transition the next output value will be equal to the average of the last Z outputs, and then the parameters of the filter will be changed.

### 2.5. Experimental Setup

**Figure 6:**
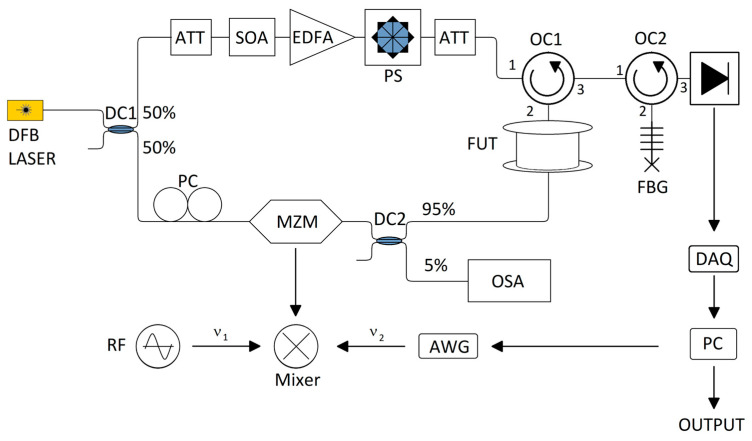
Experimental setup for the CC-BOTDA.

The experimental setup follows principles similar to the one developed in [20], shown in Figure 6: the main light source is a distributed Feedback Laser (DFB), which is split by a 50/50 directional coupler (DC1) into the pump and probe branches. The light in the pump branch is shaped into a 20 ns optical pulse (corresponding to a spatial resolution of 2 m) by intensity modulation with a high extinction ratio (>50 dB) through the use of a semiconductor optical amplifier (SOA) driven by an electrical pulse generator. The pulses are then amplified through the use of an erbium-doped fiber amplifier (EDFA), and polarization effects are removed through the use of a polarization scrambler (PS in Figure). The light in the probe branch is frequency-shifted through the use of a Mach-Zender Modulator (MZM) in a carrier-suppressed double-sideband scheme, with an extinction ratio of 20 dB and operating at null transmission point. A polarization controller (PC) is used to ensure maximum extinction ratio, which is monitored by coupling out 5% of the power through a directional coupler (DC2) to an optical spectrum analyzer (OSA). Through an optical circulator (OC1), both signals were sent into a sensing fiber with a length of ∼950 m. The modulating electrical signal sent to the MZM to create the variable probe wave is composed of 474 time-domain slots (equal to the ratio between the fiber length and the spatial resolution), with the frequency of each slot set to match the corresponding initial local BFS (which is measured through standard BOTDA and is found to be around 10.6 GHz) minus the measured Δν_op_ (30 MHz). Since the AWG maximum sampling rate is equal to 2 GHz, its output ν_1_ was limited to the 150–300 MHz range in order to maintain signal quality by staying below the Nyquist limit, and the full modulating frequency required was reached by mixing its output with the one from a fixed oscillator (RF in Figure) emitting at ν_2_ = 10.5 GHz through the use of a frequency mixer.

After experiencing the SBS amplification by the pump pulse along the sensing fiber, the probe is sent through an optical circulator OC2 into a fiber Bragg grating (FBG) where only the lower sideband component is filtered out with 6 GHz bandwidth, while the intensity of the lower sideband is detected by a 75 MHz bandwidth photodetector (PD). Afterwards, the data is sent to a data acquisition system (DAQ).

In order to suppress overall noise, every probe trace is time-averaged 512 times. For this fiber, the measurement time amounts to ∼170–200 ms. The main limitations in terms of measurement speed are the time needed for the averaging and for transmitting the updated waveform data to the AWG. It is of note that both these values could be reduced by employing a data acquisition card with integrated averaging and an AWG that can calculate the waveforms on its own from the input frequency values instead of the entire waveform data.

The AWG is controlled by a P or PI controller, linking the acquired local gains and its outputs to continuously update the probe frequency.

To test the sensing system and the filtering methods described above, a hotspot at the beginning of the fiber was immerged in a thermal bath, whose temperature was controlled by a LAUDA RM6 thermostat. Two different heating processes were tested: a step-like temperature increase, where the hotspot is immersed in pre-heated water, and a ramping process, where the hotspot is in the water as it heats up. The experimental setup was controlled through LabView 16.0, while data was analyzed with MatLab R2020b.

## 3. Results

We evaluated how employing the filtering system described in Section 3 compares in performance with the P and PI controllers chosen as optimal in [20], namely a P controller with K_P_ = 1/16 and PI controller with K_P_ = 1/16, K_I_ = K_P_/200. To do so, we performed temperature measurements featuring step-like and ramp-like temperature increases from 22 to 35 °C, over 3–4 min (equivalent to around 1000 measurement steps) in the case of the ramp.

Compared to [20], measurement time was considerably reduced (from 1.7 s to 200 ms), and irregularities in the heating process and temperature fluctuations became measurable, making every temperature ramp event different. To make measurements comparable, we applied the results from Section 2: we performed a single measurement with K_P_ = 1 and then applied the P and PI controllers as virtual filters, using Equation (2). We verified that this did not affect the measurement by performing multiple measurements both directly employing P and PI controllers and employing a controller with K_P_ = 1 whose output was then filtered by a virtual controller with the same parameters, showing similar results.

**Figure 7:**
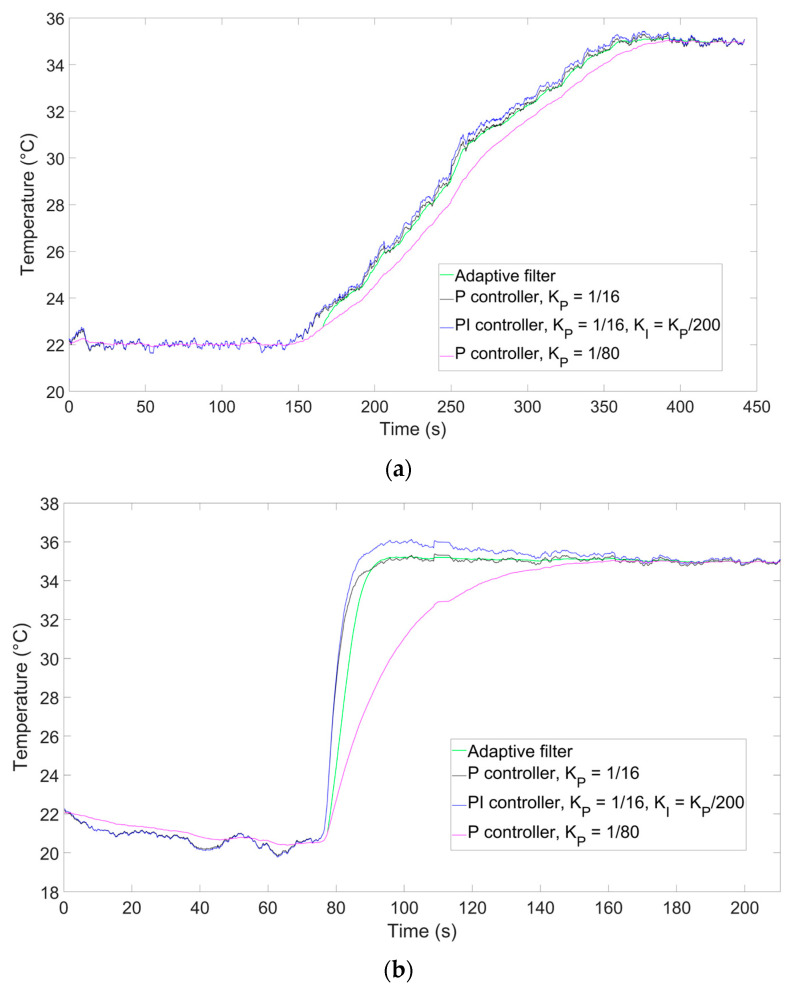
Output comparison for (**a**) a temperature ramp and (**b**) a temperature step.

The comparisons of the outputs of the adaptive filter and the P (K_P_ = 1/16) and PI (K_P_ = 1/16, K_I_ = K_P_/200) controllers are shown in Figure 7 for step-like (Figure 7a) and ramp-like processes (Figure 7b).

As shown in the Figures, the adaptive filter achieves a speed that is close to that of the P and PI controllers, albeit with a slight delay, especially compared to the PI controller. Noise levels were computed as in [20], namely as the standard deviation of the last 150 temperature readings (equivalent to the last 30 s of the experiment), when the temperature was stable at 35 °C, and when temperatures were found to be 0.101, 0.100, and 0.022 °C for the PI, P controller, and the adaptive filter, respectively, showing the greater noise suppression of the adaptive filter when the temperature is stable. For further highlighting the benefits provided by the adaptive filter, the graphs also include a reconstructed measurement with K_P_ = 1/80, which is the same as the filter used for stationary phases in the adaptive filter, which is expected to provide the same noise suppression. Indeed, the noise level for this filter was 0.021 °C, virtually identical to the adaptive filter, but its response speed is visibly slower.

**Figure 8:**
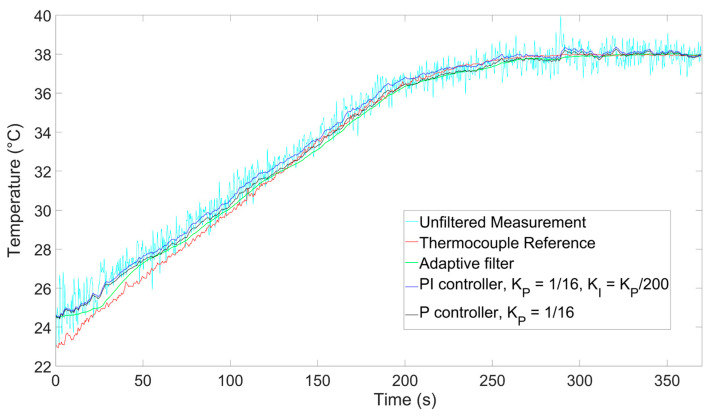
Comparison between BOTDA measurements and thermocouple during a ramping process.

To provide a better understanding of the filtering properties during the dynamic phase of the process, we have also performed an additional ramp measurement where we included a thermocouple (TC) as a temperature reference during the heating process. The results are shown in Figure 8. To estimate the uncertainty during the heating process, we calculated the root mean square error (RMSE) of the difference between the temperature measured by the TC and the one obtained as output by each filter. To calculate the RMSE, we chose the time frame between 70 and 205 s in order to capture the whole ramping phase while ruling out transient effects at the beginning and at the end of the process. We obtained RMSE values of 0.25 °C for the adaptive filter, 0.31 °C for the P controller, and 0.52 °C for the PI controller. Comparing the RMSE values of the P controller and the adaptive filter, we see a noise suppression increase of 24%, which is lower but compatible with the 30% figure we had expected in Section 2.3, while the RMSE of the PI controller is almost double.

These experimental results suggest that the adaptive filter provides better noise suppression compared to the other filters (even during the dynamic process). Note that the TC ability to accurately measure the actual fiber temperature is limited by known factors (such as temperature mismatch in the thermic bath or TC measurement noise); hence, we performed a quantitative simulative analysis also confirming the improvement in noise suppression with our adaptive filter, as will be shown below in Section 4.

**Figure 9:**
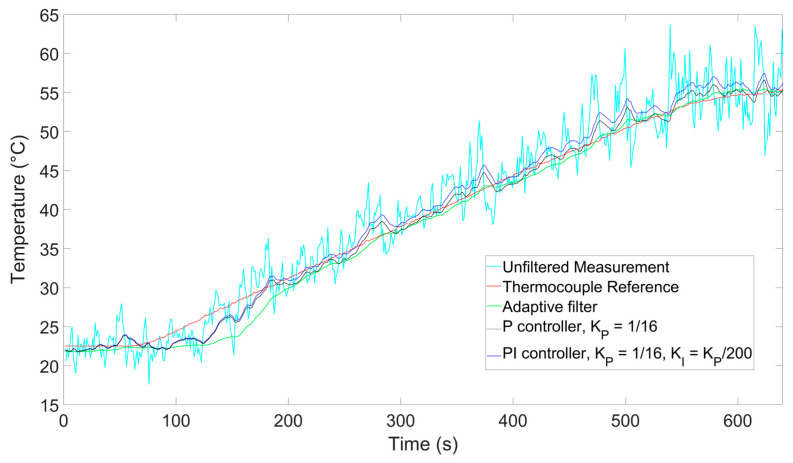
Comparison between BOTDA measurements and thermocouple during a ramping process at a sensing distance of 10 km.

As a further test to confirm the effectiveness of the system at longer distances and at greater temperature ranges, we performed a final measurement where we inserted a segment at the end of a 10 km-long fiber in a thermic bath heated up to 55 °C. The results are shown in Figure 9. Using the same method as above, we calculated the RMSE in a timeframe chosen in the middle of the ramp (between 200 and 500 s) and found values of 0.65 °C for the adaptive filter, 0.90 °C for the P controller, and 1.46 °C for the PI controller. By calculating the standard deviation of the measurements over the last 60 s, where the temperature stabilized, we found values of 0.147 °C for the adaptive filter, 0.631 °C for the P controller, and 0.635 °C for the PI controller. These results indicate that even for longer fiber lengths the adaptive filter can outperform the others. In addition, the longer heating process confirms a higher temperature measurement range for the sensing system.

## 4. Discussion

As we saw in the previous section, the experimental comparison indicated that the adaptive filter provided improved noise suppression both in the stationary and dynamic phases of the heating process. Still, experimental conditions provided limitations in the accuracy of the results, especially for estimating improvements in noise suppression for the dynamic phase.

To obtain a better view of the noise suppression from all filters at every step of the measurement, the experimental scenario detailed above was simulated in a Matlab environment in the form of a climbing temperature value with a white noise component. The parameters for noise and ramp climb rate were chosen to have a similar ratio to the ones measured in the experiment, as was the shape of the curve (two stable states with a 160 s long ramp between them, equivalent to 800 measurement steps). This curve was then passed to the virtual P and PI controllers and to the adaptive filter. In order to extract the noise at every step of the measurement, this simulation was repeated 1000 times, each time with a newly generated random noise added to the same temperature ramp. The noise level at every step for a given output was then calculated as the standard deviation of the value at the same step for each simulation.

**Figure 10:**
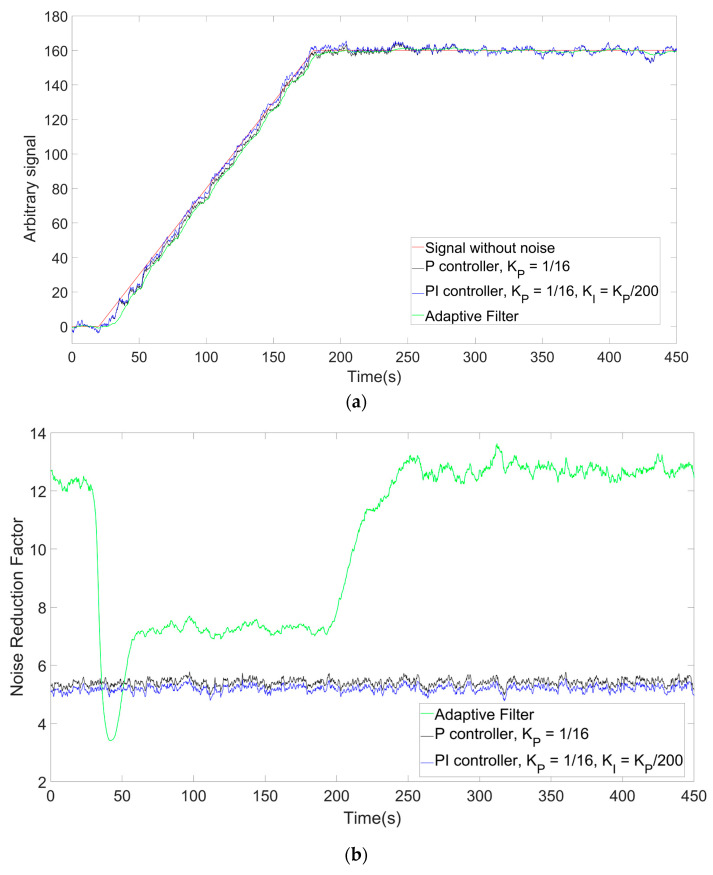
Numerical simulation of a ramp-like climb, including a comparison of the filtered ramps (**a**) and of the stepwise noise reduction factors (**b**).

An example of the outputs is shown in Figure 10a, while the measured noise suppression per each step is shown in Figure 10b. The adaptive filter noise suppression factor is 40% higher in the points corresponding to the dynamic state while, as expected from the measurements above, it is more than double in the points corresponding to the stationary phase, which is in agreement with the results obtained from the measurements. From this simulation, it can be inferred that the limited virtual PI detailed in Section 3, which was noted to have better performances in terms of standard PI in terms of overshoot and capability to follow a ramp signal, also displays better performance in terms of noise suppression. The only phase of the measurement where the adaptive filter presents lower noise suppression compared to P and PI controllers is at the beginning of the temperature ramp where, due to the increase in the signal being at first comparatively small with respect to the noise, the transition from static to dynamic states is recognized at different points in every simulation.

## 5. Conclusions

In this work, we present a digital adaptive filtering method that adapts in real time to changes in the measured environment, with the goal of improving the noise suppressing capabilities for dynamic BOTDA sensing systems while maintaining similar dynamic response speed. We implemented the system in a CC-BOTDA sensing system similar to [20], using it as a digital filter while leaving the P controller to track the evolution of the BFS.

In the experimental validation (sensing fiber in a bath of heated water) and numerical simulation, this system was compared to the optimal parameters for P and PI controllers found in [20], equal to K_P_ = 1/16 and to K_P_ = 1/16, and K_I_ = K_P_/200, respectively. The results showed that the adaptive filter had a similar response speed to the two controllers, while achieving noise suppression levels that were 40% higher than the two controllers when the temperature was changing and more than 100% higher when the temperature was stable, showing how it is possible to obtain significantly improved tradeoffs between noise suppression and response speed with this filtering method.

While in this work we focus on CC-BOTDA, the adaptive filtering system can be applied to a variety of other dynamic sensing applications acquiring continuous streams of data in the time-domain and adapting to changing conditions as measurement takes place.

In terms of tradeoff between effectiveness and a priori knowledge, this system can be seen as a middle way between simple linear filters (e.g., moving averages) and optimal nonlinear filters (e.g., Kalman filter). Unlike linear filters, the system improves performance by providing a degree of adaptability to the input data while only requiring prior knowledge on the expected environmental noise, which is a much less strict requirement compared to what is needed for Kalman filters to function, resulting in a technique capable of improving noise suppression across a wider range of applications where knowledge of the measured process would otherwise be insufficient.

## Data Availability

The raw data supporting the conclusions of this article will be made available by the authors on request.

## Abbreviations

The following abbreviations are used in this manuscript:

BGS	Brillouin Gain Spectrum
PID	Proportional Integral Derivative
BOTDA	Brillouin Optical Time-Domain Analysis
CC-BOTDA	Closed-Circuit—Brillouin Optical Time-Domain Analysis
